# Improving Ambulation and Minimizing Disability with Therapeutic Plasma Exchange in a Stiff-person Syndrome Patient with Recurrent Falls

**DOI:** 10.7759/cureus.6209

**Published:** 2019-11-20

**Authors:** Kyle Sanchez, Aqsa Ullah, Alexandria R Waler, Yassar Chakfe

**Affiliations:** 1 Medicine, University of Central Florida College of Medicine, Orlando, USA; 2 Neurology, University of Central Florida/Osceola Regional Medical Center, Orlando, USA

**Keywords:** stiff person syndrome, stiff man syndrome, therapeutic plasma exchange

## Abstract

Stiff-person syndrome (SPS) is a rare, autoimmune, neuromuscular disorder that manifests with axial and proximal muscle stiffness, rigidity, and painful muscle spasms, often causing progressive disability due to limited movement. First-line therapies comprise symptomatic management with γ-aminobutyric acid-modulating drugs such as benzodiazepines and baclofen. Patients resistant to these treatments are often given intravenous immunoglobulin (IVIg). Severe disease refractory to first-line therapy and IVIg may be treated with therapeutic plasma exchange (TPE) or immunomodulatory agents such as rituximab. Current evidence derived from case reports and case series has shown that roughly half of SPS patients treated with TPE report benefits. Here, we report the case of a 68-year-old man with a 20-year history of severe SPS and recurrent falls who was admitted to the emergency department for a traumatic hip fracture. He had significant rigidity in the axial and extremity muscles with persistent spasms of the quadriceps femoris muscle. Postoperatively, he was unable to participate in physical therapy (PT) due to these symptoms. He previously failed treatment with diazepam, baclofen, and monthly IVIg. Under our care, he underwent seven TPE treatments. By the end of treatment, he reported significant improvement in mobility with a resolution of muscle spasms and was able to be discharged to inpatient rehabilitation. This suggests that TPE may offer an effective, safe treatment modality for patients with severe refractory SPS that may significantly improve mobility and disability associated with the disease.

## Introduction

Stiff-person syndrome (SPS), previously known as stiff-man syndrome, is a rare neuromuscular disorder characterized by muscle stiffness, rigidity, and painful spasms involving the axial and proximal limb muscles [1]. The disease is likely caused by the autoimmune blockade of glutamic acid decarboxylase (GAD), an enzyme that catalyzes the synthesis of inhibitory neurotransmitter γ-aminobutyric acid (GABA). There is an elevated level of anti-GAD antibodies in the serum of ~65% of patients with SPS [2] and the disease is associated with autoimmune conditions such as type 1 diabetes mellitus, thyroiditis, and pernicious anemia [3]. A decline in the central nervous system (CNS) levels of GABA leads to a loss of neural inhibition of skeletal muscles, resulting in excessive, unintentional muscle contractions. The three subtypes of SPS include classic SPS, partial SPS, and paraneoplastic SPS, with classic SPS being the most commonly encountered variant [1].

Patients with classic SPS generally have progressive muscle rigidity and stiffness that begin in the axial muscles, especially the lumbar paraspinal muscles, and evolve over time to involve the proximal limb muscles of both upper and lower extremities [4]. The persistent muscle contractions are described as having a “board-like” quality and often cause an elevation of serum creatine kinase (CK) [5]. As truncal flexibility declines, patients commonly develop a wide-based gait and difficulty with ambulation. Classic SPS also manifests with painful episodic muscle spasms elicited by physical or emotional stress and other patient-specific triggers [2].

Most patients with classic SPS have symptomatic improvement with GABA-modulating medications such as diazepam and baclofen [5]. There is inconsistent data on the management of severe SPS resistant to conventional drug therapies. Patients with high anti-GAD antibody levels and symptoms refractory to these drugs are often given trials of intravenous immune globulin (IVIg) to neutralize the serum anti-GAD antibodies [6-7]. Therapeutic plasma exchange (TPE) [8-13], physical therapy (PT) [4,14], rituximab [15-16], cognitive behavioral therapy [17], levetiracetam [18], valproate [19], and other agents have been used in a small number of cases. There is current research interest in the efficacy and safety of using TPE over IVIg for severe SPS. Although clinical improvement in up to 56% of patients who received TPE has been shown [8], it is still reserved to treat only those cases proven to be resistant. The efficacy of TPE as a treatment for refractory SPS has not been extensively investigated, and many studies reporting its benefits have been based on patients with concurrent type 1 diabetes mellitus [10,13,20] or exclusively autoantibody-positive patients [12]. We report a case of severe SPS unresponsive to first-line medical therapies and IVIg that essentially resolved with the administration of TPE.

## Case presentation

**Figure 1:**
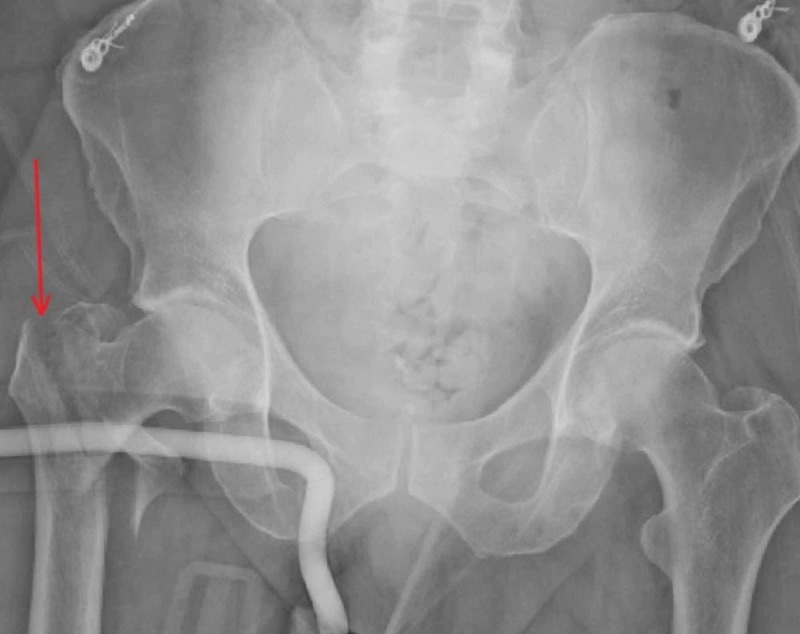
Pelvic X-ray of the displaced comminuted right intertrochanteric fracture (arrow)

A 68-year-old man presented to the emergency department (ED) with severe right thigh pain and left leg spasms after having suffered a ground-level fall onto his right side. On physical exam, he was hemodynamically stable, with minor skin abrasions on his right elbow and both lower extremities. The pelvic X-ray showed a displaced comminuted right intertrochanteric fracture (Figure 1). His right lower extremity was placed in a Hare traction splint, and he underwent an open reduction and intramedullary gamma nail fixation of the right femur. He was given baclofen (10 mg twice daily) and diazepam (10 mg twice daily) for his left leg spasms postoperatively.

With regards to his past medical history, he reported a 20-year history of stiff-person syndrome (SPS) with frequent muscle spasms and recurrent falls. He stated his doctors have been unable to adequately control his symptoms of SPS despite multiple therapies. On initial diagnosis, he was started on baclofen and diazepam with no significant relief of his spasms. He then underwent IVIg treatments three days per month for one year, followed by diazepam as maintenance therapy. He reported a mild improvement in symptoms with IVIg infusions but they did not sufficiently improve his ambulation or minimize his risk of fall. He continued to have falls, especially during physical activities such as playing tennis; one of which caused a subdural hematoma that required a left craniotomy. He remarked that he was once a high-functioning, active individual but has since been limited by his condition. 

On postoperative day one, he reported that his right thigh pain was declining, but he continued to have painful left leg spasms that worsened with movement. On physical exam, he displayed significant gait instability, decreased functional mobility, hypertonicity of his bilateral upper extremities, and prominent muscle spasms of his left quadriceps femoris muscle. His neurological examination was otherwise within normal limits. The patient’s pain from the fracture continued to improve over the second and third postoperative days.

Despite adequate recovery from the surgery, the patient continued to have marked hypertonicity of his upper extremities and painful muscle spasms of his left leg that prevented him from participating in PT. Anti-GAD antibody levels were remarkably elevated at a value >250 IU/mL (0.0 - 5.0 IU/mL) that was too high to be quantified by the laboratory. Creatine kinase (CK) levels were elevated at 469 U/L (35 - 232 U/L).

Due to his nonresponsiveness to conventional therapy and the high severity of disease, a series of seven treatments of TPE over 14 days were initiated. While discussing the treatment plan with the patient, he expressed little hope in TPE improving his condition, as he had found minimal relief after multiple trials of medications, including the year-long trial of IVIg therapy.

Over the two-week course of TPE, his functional status and symptoms of SPS significantly improved. Although his hyperreflexia remained stable at 3+, his muscle spasms and hypertonicity progressively improved after each treatment of TPE to the point that he had normal range of motion in all extremities. While receiving TPE, he was able to tolerate three hours of PT sessions daily for up to five days per week, whereas he had zero rehabilitation potential before TPE was initiated. He made significant functional gains using adaptive equipment, corrected his gait, and was eventually able to ambulate without assistance. He felt his symptoms were adequately controlled for the first time since his diagnosis and reported having no symptoms by hospital day five. Shortly after his TPE therapy was complete, he was deemed to be in a stable condition and discharged to inpatient rehabilitation.

## Discussion

SPS is a rare disorder characterized by episodic muscle pain, stiffness, and spasms [1] and is likely caused by autoantibodies to GAD with resulting disinhibition of muscle contraction [2]. Gait abnormalities, difficulty with ambulation, and recurrent falls are also common in patients with SPS, and laboratory studies often reveal elevated CK levels and high anti-GAD antibody titers [2]. First-line therapy for SPS is outpatient treatment with benzodiazepines and/or baclofen. The management of severe SPS resistant to conventional drug therapies, as in this patient, is unclear. Trials of IVIg are often given to patients who do not respond to these medications [6], especially those with high anti-GAD antibody levels [9]. However, many patients may not adequately respond to IVIg treatment, which may be due to the development of IVIG resistance, among other factors. A number of studies have documented a positive response in these patients to TPE [5,8-10] and PT sessions [4,14].

In this patient with symptoms of severe uncontrolled SPS, a high anti-GAD antibody level above 250 IU/mL, and no clinical response to GABA-modulating agents, alternative treatment with IVIg was likely the next best step in management. However, this patient’s symptoms were refractory to one year of IVIg treatment and continued to cause life-threatening problems. On two separate occasions, his recurrent falls caused a subdural hematoma and a displaced comminuted intertrochanteric hip fracture. Thus, finding an alternative treatment for his SPS was crucial, despite a lack of extensive evidence on the efficacy of many alternative treatments. After seven treatments of TPE given over 14 days, augmented by daily PT sessions up to five times per week, the patient saw a dramatic improvement in both his symptoms of SPS and functional status. His muscle spasms and hypertonicity were significantly reduced from baseline. He was able to correct his abnormal gait, gain functional mobility and ambulate without assistance. Also of note is that unlike most reported cases of SPS treated with TPE [10,13,20], this patient did not have a history of type 1 diabetes mellitus or other autoimmune conditions.

## Conclusions

This case suggests that TPE may offer an effective, safe treatment modality for patients with severe refractory SPS that may significantly improve mobility and minimize disability associated with the disease. This adds to the growing body of evidence that TPE should be offered without delay to patients who fail first-line therapy and/or IVIg.
